# The Neuroprotector Benzothiazepine CGP37157 Extends Lifespan in *C. elegans* Worms

**DOI:** 10.3389/fnagi.2018.00440

**Published:** 2019-01-17

**Authors:** Paloma García-Casas, Jessica Arias-del-Val, Pilar Alvarez-Illera, Aneta Wojnicz, Cristobal de los Ríos, Rosalba I. Fonteriz, Mayte Montero, Javier Alvarez

**Affiliations:** ^1^Department of Biochemistry and Molecular Biology and Physiology, Faculty of Medicine, Institute of Biology and Molecular Genetics (IBGM), University of Valladolid and CSIC, Valladolid, Spain; ^2^Department of Clinical Pharmacology, Instituto Teófilo Hernando, Instituto de Investigación Sanitaria la Princesa (IP), Hospital Universitario de la Princesa, Universidad Autónoma de Madrid (UAM), Madrid, Spain

**Keywords:** *C. elegans*, CGP37157, lifespan, aging, neuroprotection, Ca^2+^ signaling, mitochondria, Na^+^/Ca^2+^ exchanger

## Abstract

The benzothiazepine CGP37157 has shown neuroprotective effects in several *in vitro* models of excitotoxicity involving dysregulation of intracellular Ca^2+^ homeostasis. Although its mechanism of neuroprotection is unclear, it is probably related with some of its effects on Ca^2+^ homeostasis. CGP37157 is a well-known inhibitor of the mitochondrial Na^+^/Ca^2+^ exchanger (mNCX). However, it is not very specific and also blocks several other Ca^2+^ channels and transporters, including voltage-gated Ca^2+^ channels, plasma membrane Na^+^/Ca^2+^ exchanger and the Ca^2+^ homeostasis modulator 1 channel (CALHM1). In the present work, we have studied if CGP37157 could also induce changes in life expectancy. We now report that CGP37157 extends *C. elegans* lifespan by 10%–15% with a bell-shaped concentration-response, with high concentrations producing no effect. The effect was even larger (25% increase in life expectancy) in worms fed with heat-inactivated bacteria. The worm CGP37157 concentration producing maximum effect was measured by high-performance liquid chromatography-tandem mass spectrometry (HPLC-MS/MS) and was close to the IC_50_ for inhibition of the Na^+^/Ca^2+^ exchanger. CGP37157 also extended the lifespan in *eat-2* mutants (a model for caloric restriction), suggesting that caloric restriction is not involved in the mechanism of lifespan extension. Actually, CGP37157 produced no effect in mutants of the TOR pathway (*daf15/unc24*) or the insulin/insulin-like growth factor-1 (IGF-1) pathway (*daf-2*), indicating that the effect involves these pathways. Moreover, CGP37157 was also ineffective in *nuo-6* mutants, which have a defect in the mitochondrial respiratory chain complex I. Since it has been described that neuroprotection by this compound in cell cultures is abolished by mitochondrial inhibitors, this suggests that life extension in *C. elegans* and neuroprotection in cell cultures may share a similar mechanism involving mitochondria.

## Introduction

The benzothiazepine CGP37157 has been shown to act as a neuroprotectant in several experimental models of neurotoxicity. CGP37157 rescued neuronal death induced by veratridine in both chromaffin cells and rat hippocampal slices, with an EC_50_ of 5–10 μM (Nicolau et al., 2009, 2010). It also protected rat hippocampal slices against glutamate or ischemia/reperfusion-elicited stress (González-Lafuente et al., 2012) and SH-SY5Y human neuroblastoma cells subjected to 70 mM K^+^ stimulation (Martínez-Sanz et al., 2015). It has also been shown that CGP37157 protects primary cultures of rat cortical neurons during NMDA insults, probably through inhibition of voltage-dependent Ca^2+^ channels (VDCCs; Ruiz et al., 2014). However, it is remarkable that CGP37157 did not protect either chromaffin cells or rat hippocampal slices against the combination of the mitochondrial oxidative phosphorylation inhibitors oligomycin A + rotenone (Nicolau et al., 2010; González-Lafuente et al., 2012).

CGP37157 has been used for many years as a selective inhibitor of the mitochondrial Na^+^/Ca^2+^ exchanger (mNCX), the main mitochondrial Ca^2+^ efflux pathway. Nevertheless, a series of off-target effects of CGP37157 has been described. They include inhibition of L-type VDCC (Baron and Thayer, 1997), plasma membrane Na^+^/Ca^2+^ exchangers (Czyz and Kiedrowski, 2003), or Ca^2+^ homeostasis modulator 1 (CALHM1) Ca^2+^ channels (Moreno-Ortega et al., 2015). Many of these additional effects occur in the same range of concentrations. Therefore, it is difficult to attribute the effects of the drug to a particular molecular effect. Regarding the neuroprotective effect, it could be due to a combination of the effects CGP37157 has on different Ca^2+^ flux pathways. It has been recently shown that a designed hybrid compound of CGP37157 and the L-type Ca^2+^ channel inhibitor nimodipine had larger neuroprotective activity than any of the compounds separately when tested on *in vitro* cellular and tissue slices models related to cerebral ischemia (Buendia et al., 2017).

Many neuroprotective drugs have anti-aging activity as well (Cooper et al., 2015; Zárate et al., 2017). In the case of CGP37157, most of its known targets are also present in *C. elegans*, a well-known model for lifespan studies, and therefore a similar type of effects should be expected. Regarding the mNCX, in fact, the diversity of isoforms is much higher in *C. elegans*. Humans have only one isoform of the mNCX, named NCLX (Palty et al., 2010). Instead, *C. elegans* has 10 different isoforms (named *ncx-1* to *ncx-10*) and their functional role is not known in detail (Sharma et al., 2013; He and O’Halloran, 2014; Sharma and O’Halloran, 2014). One of them, *ncx-9*, has been reported to perform CGP37157-sensitive Na^+^/Ca^2+^ exchange activity in mitochondria (Sharma et al., 2017). *C. elegans* has also a single CALHM1 homolog, named *clhm-1* (Tanis et al., 2013). It is present in the plasma membrane of muscle cells and sensory neurons and works as well as a VDCC regulated by extracellular Ca^2+^. Knock-out of CALHM1 produces altered locomotion and its overexpression is toxic, producing degeneration through a Ca^2+^-dependent mechanism (Tanis et al., 2013). As to L-type Ca^2+^ channels, *C. elegans* has only one gene encoding an L-type α1 VDCC subunit, named *egl-19* and responsible for the action potentials in pharynx and body wall muscle (Lee et al., 1997; Jospin et al., 2002; Shtonda and Avery, 2005; Gao and Zhen, 2011; Liu et al., 2011), but the effect of CGP37157 on the *egl-19* Ca^2+^ channel has not been tested.

In the present work, we have studied the effect of CGP37157 on lifespan in *C. elegans* nematodes. Our data show that submaximal CGP37157 concentrations extended lifespan in wild-type worms and in *eat-2* mutants, a model of caloric restriction. The increase in life expectancy was even larger in worms fed with heat-inactivated bacteria. Instead, CGP37157 had no effect in mutants of two well-known nutrient-sensitive pathways: *daf-15/unc-24* (TOR signaling pathway) and *daf-2* insulin-like growth factor-1 (IGF-1 signaling pathway). CGP37157 also had no effect on *nuo-6* mitochondrial respiratory chain mutants. This reminds the lack of neuroprotective effect of CGP37157 on the neuronal death induced by mitochondrial respiratory chain inhibitors (Nicolau et al., 2010; González-Lafuente et al., 2012), and suggests that lifespan extension and neuroprotection induced by CGP37157 may occur by a similar mechanism involving mitochondria.

## Materials and Methods

### *C. elegans* Strains and Maintenance

Strains used were as follows: AQ2038, an integrated strain expressing cytosolic cameleon 2.1. (YC2.1) in pharynx controlled by the *myo-2* promoter (pmyo-2::YC2.1; Alvarez-Illera et al., 2016), kindly provided by Drs. Robyn Branicky and W. Schafer, MRC Laboratory of Molecular Biology, Cambridge, UK. Used here as a control. Its lifespan was not significantly different from that of the N2 strain (data not shown). Mutant *eat-2(ad1113), nuo-6(qm200), daf-15(m81)*/*unc-24(e138), unc-24(e138)* and *daf-2(e1370)* strains were obtained from the *Caenorhabditis* Genetics Center. Heterozygotes *unc-24(e138)/+* were obtained by crossing the *unc-24(e138)* strain with the AQ2038 strain. Worms were maintained and handled as previously described (Stiernagle, 2006). NGM agar plates were seeded with *Escherichia coli* (OP50). Strains were maintained at 20°C.

### Administration of CGP37157 to the Worms

CGP37157 is a very lipophilic drug, showing very poor water solubility (Pei et al., 2003; Martínez-Sanz et al., 2016). This property may difficult the accessibility and distribution of the drug in the worms. Thus, we have not only assayed the effect of CGP37157 dissolved in the NGM agar, but we have also used inclusion compounds with γ-cyclodextrin as a vehicle for drug administration. The γ-cyclodextrin inclusion compounds were prepared as described before (Kashima et al., 2012). Briefly, a 230 mg/ml water solution of γ-cyclodextrin was mixed 10:1 with a 50 mM DMSO solution of CGP37157 or with a 25 mM EtOH solution of cholesterol, stirred in the shaker at 1,200 rpm during 20 h and centrifuged at 12,500 rpm for 10 min. The supernatant was carefully discarded and the resulting inclusion compound was dried in the hood, weighed and dissolved in M9 buffer. The inclusion compounds in the amounts indicated were mixed with OP50 for addition to the plates.

### *C. elegans* Lifespan Assays

Eggs were obtained as described previously (Stiernagle, 2006) and transferred to *E. coli* (OP50) seeded NGM plates, either control plates or plates prepared in the presence of the required drug. For each assay, around 100 synchronized young adults (day 1) were transferred to *E. coli* (OP50) seeded NGM plates (35 mm plates, 10 worms/plate) containing 15 μM Fluorodeoxyuridine (FUdR) to avoid progeny. Control and drug-containing assays were always carried out in parallel. Plates were scored for dead worms every day. Worms that did not respond to touch with a platinum wire were considered dead. Age refers to days following adulthood. Plates with fungal contamination during the first 10 days of the assay were excluded from the study. Missing worms, individuals with extruded gonad or desiccated by crawling in the edge of the plate were censored, as well as plates with fungal contamination after the first 10 days. Control and drug-containing plates were kept close together in a temperature-controlled incubator set at 20°C. Statistical analysis was performed with the SPSS software, using the Kaplan-Meier estimator and the log-rank routine for significance.

For the experiments with dead bacteria, OP50 were grown overnight at 37°C and then heat inactivated at 65°C for 30 min. After treatment, an aliquot was found not to grow when placed in LB medium. NGM plates were then seeded with heat-killed bacteria and the rest of the assay was as described above.

### *C. elegans* Fertility Measurement

Freshly OP50 seeded NGM plates were prepared without FUdR and with or without 50 μM CGP37157. Then, 1 L4 larva was transferred to each plate and the number of eggs laid was counted every 24 h after transferring the worm to a new plate.

### Measurement of CGP37157 in *C. elegans* Worms

Around 4,000 *C. elegans* worms were transferred at day 1 of adulthood to treatment plates containing 1 μg of CGP37157-containing γ-cyclodextrin inclusion compound mixed with OP50. At day 8 of treatment, worms were collected and washed by centrifugation at 2,000 rpm with cold water. The supernatant was removed and 500 μL of cold methanol was added. Worms were resuspended and sonicated during 50 cycles (5 s on/5 s off). The suspension obtained was centrifuged at 5,000 rpm for 5 min. The supernatant was collected and stored at −80°C until its analysis by high-performance liquid chromatography-tandem mass spectrometry (HPLC-MS/MS).

### Determination of CGP37157 From *C. elegans* Extracts by HPLC-MS/MS

CGP37157 quantification was performed using a HPLC-MS/MS system. The instrument consisted of HPLC, 1200 Series separation module (Agilent Technologies, Santa Clara, CA, USA) coupled to triple quadrupole mass spectrometer (MS/MS, 6410 series) equipped with electrospray ionization source (ESI). HPLC-MS/MS system was controlled by Agilent Mass Hunter Workstation Data Acquisition software. The MS/MS was operating in positive multiple reaction monitoring mode and the conditions were set as followed: desolvation gas (N_2_) flow 12 L/min, nebulizer pressure 60 psi, drying gas temperature 300°C and capillary voltage 4,000 V. The *m/z* ratios for the CGP37157 quantifier and qualifier ions were 324.1 > 214.1 and 324.1 > 179.1, respectively. The HPLC separation was carried out at 25°C in a reversed-phase C18 column (ZORBAX Eclipse XDB, 4.6 mm × 150 mm and 5 μm particle size; Agilent Technologies, Santa Clara, CA, USA) protected by a 0.2-μm on-line filter. 0.2% formic acid in water, pH = 3.0 (A) and 0.2% formic acid in ACN (B; 30:70, v/v) were used as the mobile phase. The chromatogram was run under gradient conditions at a flow rate of 0.8 mL/min. The following gradient program was used for CGP37157 separation: 70% of B at 0.0–0.5 min; gradually increasing phase B to 100% at 0.5–1.0 min; 100% of B at 1.0–2.0 min; returning to the initial conditions (30% of A and 70% of B) at 2.0–2.5 min; followed by a re-equilibration time of 2.5 min, to give a total run time of 5 min. Five μL of CGP37157 was injected into the chromatographic system.

### Materials

CGP37157 was synthesized as previously described (Martínez-Sanz et al., 2015). γ-cyclodextrin was purchased from PanReac, Barcelona, Spain. FuDR was acquired from Alfa Aesar, Karlsruhe, Germany. Other reagents were obtained from Sigma, Madrid, Spain or Merck, Darmstadt, Germany.

## Results and Discussion

Because of the poor water solubility of CGP37157, we have used two different methods of drug administration to the nematodes. First, we used γ-cyclodextrin to generate an inclusion compound which is then added to the NGM plates together with the OP50. In this way, the drug is ingested by the worms together with the OP50 and directly absorbed into the intestine. Once prepared as described in “Materials and Methods,” the inclusion compound is weighed and dissolved in M9 buffer, so that a known amount (in μg of inclusion compound) is added to the plates. The second method was to directly dissolve the compound in NGM agar at the maximum possible concentration, which was 25–150 μM.

**Figure 1:**
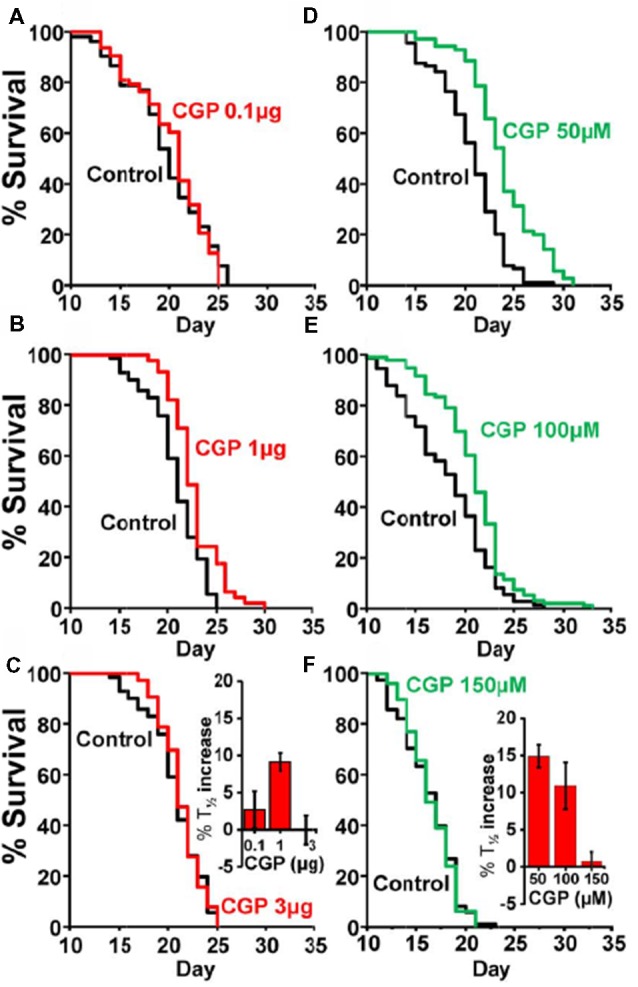
Effects of CGP37157 on survival in *C. elegans*. Panels **(A–C)** show representative survival plots corresponding to parallel lifespan assays performed using γ-cyclodextrin-inclusion compounds in the following conditions: Control/CGP37157 (**A**: 0.1 μg; **B**: 1 μg; **C**: 3 μg). Panels **(D–F)** show representative survival plots from Control/CGP37157 lifespan assays in which either 50 μM, 100 μM or 150 μM CGP37157 were dissolved in the NGM agar. The assays shown correspond to those marked in bold in Table 1. The insets placed in panels **(C,F)** show the mean increase in survival obtained in several similar lifespan assays of each kind (more details of all the assays in Table 1).

Table 1 shows the results of a series of lifespan assays performed with several concentrations of CGP37157, added by any of the two methods to wild-type worms. Figure 1 shows plots of typical lifespan assays obtained for each condition. The plots correspond to the assays labeled in bold in Table 1. Figures 1A–C show the effect of three different amounts of the inclusion compound, 0.1, 1, and 3 μg. Figures 1D–F show the effect of three concentrations of CGP37157 (50, 100 and 150 μM) directly dissolved in NGM agar. Figure 1F summarizes the mean increases in lifespan obtained. In the case of the inclusion compound, the maximum lifespan extension was obtained at 1 μg, which increased lifespan by nearly 10%. Concentrations below or above that level produced a much lower effect or even no effect. Direct effects of γ-cyclodextrin on *C. elegans* lifespan were excluded by studying the effect of a γ-cyclodextrin-cholesterol inclusion compound, which produced no effect (Table 2). Moreover, similar or even larger effects were obtained when CGP37157 was directly added to the NGM agar, showing that the effect was not dependent on the administration pathway. Both 50 and 100 μM CGP37157 extended lifespan by 10%–15% with high statistical significance, but again here increasing the concentration to 150 μM produced no effect. A lower concentration, 25 μM, was also ineffective (see Table 1). Therefore, CGP37157 is able to extend the *C. elegans* lifespan in a certain concentration range, suggesting that submaximal inhibition of one or more of their targets is required.

**Table 1:** Treatment of wild-type worms with CGP37157.

Drug	Lifespan drug (days)	*N* Drug	Lifespan control (days)	*N* Control	% Lifespan increase	*P* value Drug vs. Control	Mean % lifespan increase
CGP 0.1 μg	18.6	63/76	17.0	69/81	9.6	<0.0001	**2.7 ± 2.5**
	20.5	78/100	19.3	62/80	6.4	<0.003
	**20.7**	**63/80**	**20.1**	**52/64**	**3.0**	**0.636**	
	20.7	78/98	21.0	71/95	−1.7	0.668
	21.4	45/60	22.3	78/103	−3.9	<0.024
CGP 1 μg	19.3	88/101	17.0	69/81	13.4	<0.0001	**9.1 ± 1.2**
	20.9	72/83	19.3	62/80	8.4	<0.001
	21.9	48/61	20.1	52/64	9.4	0.092
	**22.8**	**45/52**	**21.0**	**71/95**	**8.4**	**<0.001**
	23.7	79/103	22.3	78/103	6.0	<0.0001	
CGP 3 μg	17.6	73/99	17.0	69/81	3.8	0.112	**0.0 ± 2.0**
	20.0	51/62	19.3	62/80	3.4	0.367
	19.6	49/61	20.1	52/64	−2.2	0.962
	**21.4**	**76/101**	**21.0**	**71/95**	**1.6**	**0.86**
	20.9	49/62	22.3	78/103	−6.6	<0.008	
CGP 25 μM	22.7	86/107	22.6	82/100	0.6	0.815	**0.6**
CGP 50 μM	25.6	90/107	22.9	82/100	11.9	<0.0001	**14.9 ± 1.5**
	**24.1**	**70/96**	**20.7**	**89/106**	**16.5**	**<0.0001**	
	21.4	105/114	18.4	74/80	16.3	<0.0001
CGP 100 μM	24.7	94/104	22.9	82/100	7.8	<0.0001	**10.9 ± 3.1**
	**21.0**	**96/105**	**18.4**	**74/80**	**14.0**	**<0.0001**	
CGP 150 μM	**16.6**	**79/90**	**16.4**	**85/92**	**1.2**	**0.994**	**0.8 ± 1.2**
	16.7	99/104	17.1	85/104	−2.1	0.073
	15.2	95/101	15.1	94/103	0.5	0.98
	16.0	94/103	15.4	96/101	3.7	0.348

*Lifespan assays performed with CGP37157 in wild-type worms. The table shows the drug concentration used in each series of assays, the half-life ($T\frac{1}{2}$) of the worms incubated with the drug obtained from the Kaplan-Meier analysis, the number of worms in the drug-containing assay (final/total), the half-life ($T\frac{1}{2}$) of the control worms, the number of worms in the control assay (final/total), the % increase in the half-life, the statistical significance of the difference between control and treated worms, obtained from the log-rank test, and the mean ± SE increase in half-life from all the series made with the same drug concentration. Concentrations in μg for γ-cyclodextrin-inclusion compounds and in μM for compound dissolved in NGM agar. In bold, series shown in the survival plots of Figure 1*.

**Table 2:** Control experiments: effect of γ-CD-cholesterol and effect of treatment with CGP37157 in wild-type worms fed with dead OP50.

Drug	Lifespan drug (days)	*N* Drug	Lifespan control (days)	*N* Control	% Lifespan increase	*P* value Drug vs. Control	Mean % lifespan increase
**A: Wild-type worms. Effect of γ-CD-cholesterol**							
γ-CD-chol10 μg	16.3	75/91	16.4	85/92	−0.54	0.95	**−0.5 ± 0.8**
	16.9	84/91	17.1	85/104	−0.94	0.598
	14.8	85/101	15.1	94/103	−2.32	0.414
	15.6	80/93	15.4	96/101	1.65	0.996
**B: Wild-type worms fed with dead OP50. Effect of CGP37157**							
CGP 50 μM	**23.2**	**71/98**	**17.7**	**56/74**	**31.0**	**<0.0001**	**25.6 ± 1.9**
	25.4	69/100	20.5	80/100	24.1	<0.0001
	18.8	66/100	15.4	75/100	22.2	<0.0001
	21.3	63/80	17.0	46/60	25.0	<0.0001

*Control lifespan assays. Part A: effect of the drug carrier γ-CD, studied using a γ-CD-cholesterol inclusion compound. Part B: effect of 50 μM CGP37157 in wild-type worms fed with dead OP50. The table shows a series of lifespan assays performed in the presence or in the absence of either γ-CD-cholesterol (A) or CGP37157 (B). Other details as in Table 1*.

To exclude the possibility that the effect of CGP37157 could be the result of a secondary metabolite elicited by bacterial action on this compound, we have studied the effect of this compound on the lifespan of *C. elegans* worms fed with heat-inactivated OP50. The results are in Table 2. CGP37157 was still able to increase the *C. elegans* lifespan under these conditions. In fact, the effect was much larger, producing a mean increase in survival above 25%. This suggests that bacterial metabolism of this compound could be interfering with its effect.

**Figure 2:**
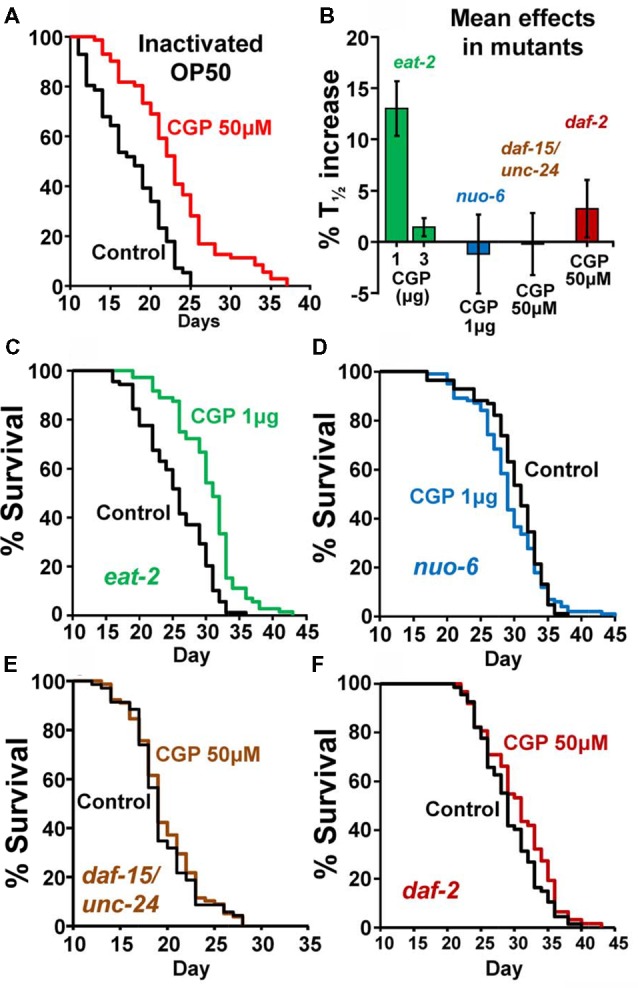
Effects of CGP37157 on survival in worms fed with inactivated OP50 or in several *C. elegans* mutants. Panel **(A)** shows the effect of 50 μM CGP37157 on the lifespan of wild-type worms fed with heat-inactivated bacteria. Panel **(B)** shows the mean effects of CGP37157 on the life expectancy of several mutant strains. Panels **(C,D)** show representative survival plots from parallel lifespan assays performed in *eat-2* and *nuo-6* mutants, respectively, using 1 μg of CGP37157 γ-cyclodextrin-inclusion compound. Panels **(E,F)** show representative survival plots from parallel lifespan assays performed in *daf15/unc24* and *daf-2* mutants, respectively, using 50 μM of CGP37157 dissolved in NGM agar. The plots correspond to the assays marked in bold in Table 3 (more details of all the assays in Table 1).

To investigate the mechanism of the lifespan extension induced by CGP37157, we have used several *C. elegans* mutants: *eat-2(ad1113), nuo-6(qm200), daf-15(m81)*/*unc-24(e138), unc-24(e138)* and *daf-2(e1370)*. Mutant *eat-2* has a defect in pharyngeal pumping that reduces the rate of feeding. This produces an increase in the survival of the mutant worms and is considered to be a model for the effects of caloric restriction (Lakowski and Hekimi, 1998). Therefore, if the effects of CGP37157 would be mediated by caloric restriction, we would expect it to produce little or no effect in *eat-2* mutants. However, when we added the CGP37157 inclusion compound to *eat-2* mutants, it produced the same effects than in wild-type worms, it increased by nearly 15% the lifespan at 1 μg, and produced no effect at 3 μg. Table 3 shows the results of a series of lifespan assays performed with these two CGP37157 concentrations in *eat-2* mutants and panels **A,B** in Figure 2 show plots of typical assays performed at each concentration. The lifespan was longer in the *eat-2* mutants than in the wild-type worms (compare with Table 1), and 1 μg of CGP37157 increased it further by nearly 15%. Instead, 3 μg of the compound had no effect, similarly to wild-type worms. These data suggest that the lifespan extension induced by CGP37157 is not due to caloric restriction.

**Table 3:** Treatment of *eat-2*, *nuo-6*, *daf-15/unc-24*, *unc-24* and *daf-2* worms with CGP37157.

Drug	Lifespan drug (days)	*N* Drug	Lifespan control (days)	*N* Control	% Lifespan increase	*P* value Drug vs. Control	Mean % lifespan increase
**eat-2**							
CGP 1 μg	**30.4**	**72/93**	**25.9**	**89/107**	**17.6**	**<0.0001**	**13.0 ± 2.7**
	26.8	67/101	24.7	88/121	8.3	<0.0001
	26.1	110/123	23.1	79/84	13.1	<0.0001
CGP 3 μg	26.6	57/97	25.9	89/107	2.9	0.696	**1.4 ± 0.9**
	**24.7**	**94/124**	**24.7**	**88/121**	**−0.1**	**0.832**
	23.5	89/105	23.1	79/84	1.5	0.453
**nuo-6**							
CGP 1 μg	34.8	117/140	32.7	115/151	6.3	<0.021	**−1.2 ± 3.8**
	**30.0**	**101/141**	**31.0**	**84/115**	**−3.3**	**0.166**
	33.1	52/100	35.4	27/55	−6.6	0.197
**daf-15/unc-24**							
CGP 50 μM	19.0	82/101	18.2	81/100	4.0	0.069	**−0.2 ± 3.0**
	**19.9**	**78/90**	**19.6**	**69/91**	**1.4**	**0.701**
	18.9	82/101	20.2	82/93	−6.1	<0.031
**unc-24/+**							
CGP 50 μM	25.0	86/96	16.7	62/75	49.5	<0.0001	**29.4 ± 10.1**
	18.7	89/99	15.7	81/97	18.9	<0.0001
	19.0	125/140	15.8	105/126	19.8	<0.0001
**daf-2**							
CGP 50 μM	**31.2**	**62/101**	**29.4**	**67/91**	**6.1**	**<0.024**	**3.3 ± 2.9**
	25.8	92/148	25.7	75/146	0.4	0.628	

*Lifespan assays performed with CGP37157 in several mutant *C. elegans* strains. The table shows a series of lifespan assays performed in several *C. elegans* mutant worms in the presence or in the absence of CGP37157. Concentrations producing the maximum effect in the controls were used, either 1 μg of γ-cyclodextrin-inclusion compound or 50 μM of the compound dissolved in NGM agar. In bold, series shown in the survival plots of Figure 2. Other details as in Table 1*.

Then, we tested the effect of the maximally effective amount of CGP37157 inclusion compound, 1 μg, on *nuo-6* mutants. These mutants have a defect in a subunit of complex I of the mitochondrial respiratory chain, and show reduced mitochondrial function, lower oxygen consumption, slow growth and movement (Yang and Hekimi, 2010b) and decreased ATP levels (Yee et al., 2014). This is accompanied by a significant lifespan extension, underscoring the importance of mitochondrial metabolism in survival (Yang and Hekimi, 2010b). In these mutants, however, we could not find any significant effect of GCP37157 on lifespan. Table 3 shows the results of a series of lifespan assays performed with 1 μg of CGP37157 in *nuo-6* mutants, and panel **C** in Figure 2 shows one of the lifespan assays. The lack of effect of CGP37157 in *nuo-6* mutants reminds the lack of neuroprotective activity CGP37157 has in cellular models treated with mitochondrial oxidative phosphorylation inhibitors (Nicolau et al., 2010; González-Lafuente et al., 2012). Although the mechanism of the effects of CGP37157 in both cases is still unknown, it is clear that both effects require functional mitochondria to develop. In this respect, we should note that the only known mitochondrial target of CGP37157 is the mNCX. Therefore, this exchanger could play a role in these effects.

In the case of the *daf-15/unc-24* mutants, as with *nuo-6* mutants, we tested the effect of 50 μM CGP37157 and we found no effect. The lack of effect was due to the DAF-15 mutation, because CGP37157 extended the lifespan of *unc-24/+* mutants as much as in the controls (Table 3). *daf-15* (raptor) heterozygous mutants have a partial suppression of the TOR pathway that produces an increase in lifespan of 13% (Jia et al., 2004). The lack of effect of CGP37157 in these mutants suggests that this pathway is somehow involved in the increase in life expectancy induced by this compound. Genetic and pharmacological inhibition of the TOR pathway has been shown to extend lifespan in many organisms, including *C. elegans* (Hansen et al., 2007). However, mitochondrial ROS-dependent TOR signaling has also been shown to be necessary for the lifespan extension induced by hypoxia (Schieber and Chandel, 2014). Given that mitochondrial Ca^2+^ is a critical regulator of ROS production (Görlach et al., 2015), changes in mitochondrial Ca^2+^ induced by CGP37157 could act on TOR signaling in this way. However, much further work is necessary to clarify this point.

Finally, our data also show that CGP37157 has little or no effect on *daf-2* mutants. The *daf-2* gene encodes for the insulin-like growth factor 1 (IGF-1) receptor, one of the best known nutrient-sensitive signaling pathways controlling lifespan (Gami and Wolkow, 2006). The lack of effect of CGP37157 in both *daf-15/unc-24* and *daf-2* mutants is consistent with the overlap that exists between both the TOR and the insulin/IGF-1 signaling pathways. The expression of DAF-15 (raptor) is negatively regulated by DAF-16, a FOXO transcription factor that is in turn negatively regulated by *daf-2* insulin/IGF-1 signaling. *daf-15* (raptor) transcription is therefore regulated by *daf-2* insulin/IGF signaling (Jia et al., 2004; Lapierre and Hansen, 2012). In addition, ROS have also been reported to be very important mediators for the increase in lifespan of *daf-2* mutants (Zarse et al., 2012; Senchuk et al., 2018).

To estimate the long-term stability of CGP37157 in the assays, as well as the effective concentration attained by the drug inside the worms when it induces lifespan extension, we have determined the concentration of CGP37157 in the worms at day 8 of adult life during a typical lifespan assay with 1 μg of the inclusion compound. Worm extracts were obtained twice, and CGP37157 was measured in triplicate samples of each extract. Considering a mean worm volume of 5 nl (So et al., 2011), the concentration values obtained were 1.48, 1.45, and 1.57 μM (extract 1) and 2.04, 2.18, and 2.16 μM (extract 2). The mean value was 1.81 ± 0.14 μM. This value is close to the IC_50_ for the inhibition of the mNCX by CGP37157 (Hernández-SanMiguel et al., 2006), although other targets of CGP37157 are also inhibited in the same concentration range. As mentioned in the Introduction, all the known targets of this compound participate in Ca^2+^ homeostasis, and it may seem reasonable to suggest a role for Ca^2+^ signaling in the mechanism of its effects. However, at this moment we cannot conclusively establish this point. In any case, the presence of CGP37157 in day 8 worms shows that the compound is stable under our experimental conditions and reaches concentrations in worms able to have a continuous submaximal inhibitory effect on some of its known targets. Regarding the assays performed with 50–150 μM CGP37157 dissolved in NGM agar, we have to consider that the drug concentrations in NGM agar generally required to produce effects in *C. elegans* are 10–100 times higher than in cell cultures.

**Figure 3:**
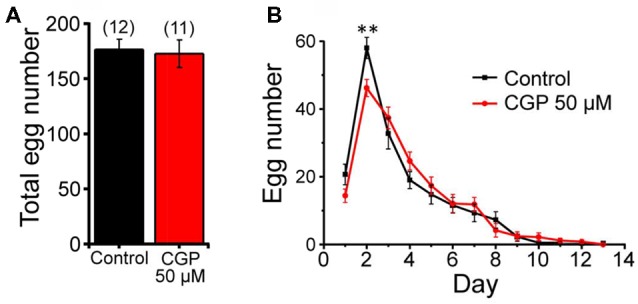
Effects of CGP37157 on egg laying. Panel **(A)** shows the total number of eggs laid per worm (mean ± SE) in the absence or in the presence of 50 μM CGP37157. Panel **(B)** shows the distribution of eggs laid per day of adult life (mean ± SE). The differences were not significant, except for day 2 (**p < 0.01).

We have also studied the possible effect of CGP37157 on *C. elegans* fertility by counting the number of eggs laid per worm every 24 h, either in the presence or in the absence of 50 μM CGP37157. Figure 3A shows that the compound had no significant effect on the total number of eggs laid. We could observe, however, that laying of eggs took place with a small delay in the worms treated with the compound, although the difference was statistically significant only at day 2 of adult life (Figure 3B). A similar trend was observed after that in both groups.

Our results show a novel correlation between neuroprotective activity in cell cultures and lifespan extension in the *C. elegans* model for the benzothiazepine CGP37157, both effects requiring functional mitochondria to develop. Another example of this correlation is the neuroprotection and lifespan extension induced by partial pharmacological uncoupling of mitochondrial oxidative phosphorylation. Mitochondrial depolarization has been proposed to be a potential therapeutic strategy for several human disorders that involve metabolic and mitochondrial oxidative stress, including Parkinson’s and Alzheimer’s diseases (Wu et al., 2011; Geisler et al., 2017), cerebral ischemia (Korde et al., 2005) and heart ischemia (Brennan et al., 2006). At the same time, partial mitochondrial uncoupling attenuates age-dependent neurodegeneration and increases survival in *C. elegans* (Lemire et al., 2009; Cho et al., 2017). Thus, mild mitochondrial dysfunction increases lifespan and triggers neuroprotection, perhaps through a hormetic response (Haigis and Yankner, 2010; López-Otín et al., 2013).

The role of mitochondrial dysfunction in aging is complex. There is a growing body of evidence suggesting that impaired mitochondrial function may protect against aging and age-associated diseases. In *C. elegans*, a large number of respiratory chain loss of function mutants have been studied, and many of them show higher lifespan (Yang and Hekimi, 2010b; Munkácsy and Rea, 2014). The reason for this paradoxical effect is still under debate (Dancy et al., 2014; Maglioni et al., 2014; Munkácsy and Rea, 2014; Hekimi et al., 2016). In the case of *nuo-6* mutants, it was found that a mild elevation of mitochondrial ${\text{O}}_{2}^{-}$ was both necessary and sufficient for the increase in life expectancy (Yang and Hekimi, 2010a; Schaar et al., 2015). The idea is that moderate elevations in ROS may trigger compensatory responses which finally lead to prolonged lifespan. The fact that CGP37157 does not increase life expectancy in *nuo-6* mitochondrial respiratory chain mutants suggests that lifespan extension by this compound may use a similar pathway. A possible mechanism could involve partial mitochondrial Na^+^/Ca^2+^ exchanger inhibition, leading to mitochondrial Ca^2+^ accumulation and increased ${\text{O}}_{2}^{-}$ production. Elevated ROS have also been shown to be very important for the increase in lifespan in *daf-2* mutants (Zarse et al., 2012; Senchuk et al., 2018) and they modulate TOR signaling (Schieber and Chandel, 2014), This could explain why CGP37157 is not effective in mutants of these pathways, but further work will be necessary to clarify the molecular mechanism of its effect.

## Data Availability

The raw data supporting the conclusions of this manuscript will be made available by the authors, without undue reservation, to any qualified researcher.

## Author Contributions

MM and JA designed the project. PG-C performed most of the lifespan experiments, and JA-V and PA-I joined in performing some of them. CR and AW made the synthesis and HPLC-MS/MS measurements of CGP37157. JA wrote the manuscript. RF, MM, CR, and AW helped in discussing and editing the manuscript. All authors read and approved the final manuscript.

## Conflict of Interest Statement

The authors declare that the research was conducted in the absence of any commercial or financial relationships that could be construed as a potential conflict of interest.
